# Crowdsourcing the CTSA Innovation Mission

**DOI:** 10.1111/cts.12147

**Published:** 2014-03-21

**Authors:** Maninder Kahlon, Leslie Yuan, Oksana Gologorskaya, S. Claiborne Johnston

**Affiliations:** ^1^ Clinical & Translational Science Institute & Department of NeurologyUniversity of California, San FranciscoSan FranciscoCaliforniaUSA; ^2^ Clinical & Translational Science InstituteUniversity of CaliforniaSan FranciscoCaliforniaUSA; ^3^ Clinical & Translational Science Institute & Department of NeurologyUniversity of CaliforniaSan FranciscoCaliforniaUSA

## CTSAs and the Mandate to Innovate

To fully meet their mission, Clinical and Translational Science Awards (CTSAs) cannot rely on old models for supporting research. Sure, providing services that investigators have come to expect is a critical role for CTSAs, but current systems of supporting research are obviously broken, with costs only increasing and barriers to delivering health improvements growing over the past several decades. Clearly, new models for supporting and executing clinical and translational research are required.

Top‐down approaches to innovation have several advantages, including assuring alignment with institutional goals and matching ideas with the organizational structure that can achieve them. Control brings simplicity and reduces risk of implementation. However, top‐down innovation may be out of touch with the needs of investigators, ultimately producing services that are underutilized. Programs may be less innovative because fewer are contributing ideas. Finally, failing to engage end‐users throughout the process may lead some academics to be less open to participation. Leaders in community engagement have recognized these risks for many years.

Traditional bottom‐up approaches for innovation, such as through requests for proposals (RFPs), are an attractive alternative. Investigators are accustomed to putting forward their best ideas for the possibility of funding, so engagement is intuitive to them through this mechanism. Ideas can come from a large number of people and the community of those working on solutions can increase. However, traditional bottom‐up approaches have limitations. Without iterative feedback, an opportunity to improve ideas is lost. Hiding proposals from other submitters eliminates the potential for new teams to come together. Also, treating such ideas as fully owned by the proposer prevents integration of similar proposals or leveraging of existing programs and personnel.

### A better bottom‐up approach

We decided to create a mechanism to regularly infuse the Clinical and Translational Science Institute (CTSI) at the University of California, San Francisco, with fresh ideas for new initiatives. Recognizing that the RFP approach engendered inefficiencies as described above, we decided to rework the process. First, we decided to open up the submission and review process, so that all contributors could see each other's proposals and identify synergies. Second, we allowed for commenting on individual proposals and iteration of proposals to incorporate improvements or new team members. Finally, we pushed for the most open approach to commenting possible, with viewing and commenting allowed by anyone, anywhere. By framing the process as a public one, we hoped to incrementally acculturate our community to this new approach to sharing proposals. Through practice we wanted to introduce the net benefits of openness even if it came with the risk of losing full control over one's initial idea. We also wanted to remove all barriers to participation for those interested. Even one password or a VPN requirement, we knew, would reduce participation. The entire process was enabled by an online “Open Proposals” platform that implemented these features, built off the popular open source Drupal software.

### Continuous innovation at UCSF's CTSI

**Figure 1:**
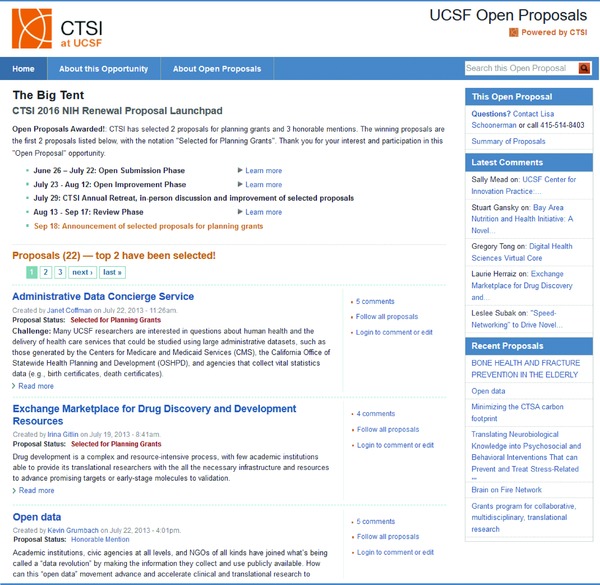
An example Open Proposals–supported RFP to solicit ideas for the CTSA renewal grant.

Over the past four years, we used evolving versions of the “Open Proposals” platform to solicit proposals for eight opportunities to add to our portfolio of activities to improve research resources at the university (Table 1A). For each of these opportunities, proposers submitted short (one page) proposals using a structured template that included an articulation of the perceived need, potential partners and impact (Figure 1). We solicited proposals and comments from a wide community using campus‐wide staff and academic listservs to invite participation. Proposers could revise their applications based on input as many times as they wanted, with the system keeping track of revisions. Each revision could in turn receive new comments. This period of ‘Open Improvement' lasted for 2–4 weeks, after which the RFP was closed. Subsequently, a more traditional peer‐review process by a committee (usually CTSI leadership) selected the best final proposal/s and teams for support and funding.

**Table 1:** Open Proposals‐enabled RFPs conducted since 2009. (A). RFPs launched and managed by UCSF CTSI. (B) RFPs launched and managed by other entities within UCSF. (C) RFPs launched and managed by other institutions.

	Audience targeted	#Proposals submitted	#Comments	Average comments per proposal	#Revised proposals	#Withdrawn proposals
(A)CTSI usage						
CTSI year 4 funding priorities, 2009—10	35	18	41	2.28	2	n/a*
CTSI stimulus/ARRA Grants Selection, 2009—2010	300	27	39	1.44	3	n/a
CTSI Renewal, 2009—2010	7500	53	47	0.89	8	n/a
CTSI Admin Supplement Selection, 2010—2011	300	19	6	0.32	4	n/a
CTSI OpenSocial Gadget Contest 2012	>1000	23	94	4.09	6	0
CTSI Translational Innovation Awards, 2011–12	>7500	28	290	10.36	22	1
CTSI Translational Innovation Awards, 2012—13	>7500	27	216	8.00	23	0
CTSI Big Tent: 2016 Renewal Launchpad	>7500	22	68	3.09	10	0
(B) External non‐CTSI usage at UCSF						
IT Innovation Contest 2012	>10000	48	216	4.50	37	1
IT Innovation Contest 2013	>10000	17	117	6.88	14	1
Dept. of Medicine, Funding Patient Cohorts	620	25	38	1.52	12	1
HR New Process Map Review	150	14	2	0.14	14	0
Provosts Office, Funding Cores, Yr 1	>5000	35	72	2.06	22	0
Provosts Office, Funding Cores, Yr 2	>5000	23	77	3.35	10	0
Center for Healthcare Value, Caring Wisely Initiative*	>10000	20	46	2.30	7	0
Coursera Course Selection	>10000	25	16	0.64	22	0
School of Medicine Deans Office, Bureaucracy Busters	>5000	39	111	2.85	12	0
Academy of Med Educators, Innovations Funds for Educators	>5000	64	331	5.17	53	5
Provosts Office, Shared Research Facilities Roadmap	>5000	61	Ongoing			
(C) External non‐UCSF usage						
UC Merced, Strategic Academic Focusing Initiative	Ongoing					
Harvard Medical School, Harvard Longwood Campus IT Day	Ongoing					

*n/a = could not measure.

All these CTSI‐managed opportunities included incentives for proposers, but in early years, these were generally nonmonetary. A call to help sculpt Year 4 activities or contribute to the first CTSI renewal grant provided indirect incentives such as the possibility that the proposer could see the project implemented (thereby addressing their need) or perhaps being involved in the process to implement the project (thereby providing them with a leadership role in a program at CTSI). Then, over the past 2 years we launched an annual RFP in which we set aside a small pool of “Innovation” funds to support new ideas from the broadest possible community at UCSF. The Innovation RFP incents proposers with potential funding to lead and implement a project that addresses what they see as a key need to improve how research is conducted. Each year CTSI leadership selected 2–4 initiatives, each receiving funding between $15K–75K. Ideas ranged from implementing an easier IRB approval process for research involving chart reviews (from an investigator who was not a part of CTSI's program leadership) to supporting a video‐based introduction to case studies in early translational research (from a senior manager in one of CTSI's programs). These are both now blossoming into larger projects; the first as an element of a broader overhaul of process improvement for IRB reviews, and the latter seeking funding from external sources to further bolster initial success.

### Using “Open Proposals” at UCSF and beyond

Over the past year, CTSI's use of the Open Proposals platform at UCSF garnered attention from others at the University and beyond. Within the University, groups ranging from central IT to individual departments and educational programs reached out to try the approach (Table 1B). In large part we were able to work with stakeholders and administrators to convey best practices. In most cases, audiences were engaged with numerous comments per proposal. CTSI‐led RFPs, especially the ‘Innovation' calls clearly did far better than others; we attribute this to our audience getting accustomed over the years to our use of this process and to carefully crafted communications. We invest in upfront seeding of the forums by CTSI leadership; our board and executive committee participate and set the tone for interactions. We also reach a very broad audience that includes students, staff and trainees, in addition to faculty. In fact, one of the most commented proposals came from a student, and generated more than 50 comments from faculty and students. Finally, unlike initiatives led by others, we consistently keep our forums public. But regardless of differences in number of comments, overall, stakeholders have provided positive feedback on engagement and the ideas generated. Two other institutions have now adopted the tool and process; Harvard Medical School is using the approach to solicit ideas for IT innovation, and UC Merced is gathering and improving ideas for the school as a whole. The tool is easily skinned to promote the offering institution.

Average comments received per proposal is a measure of engagement of the broad community in the Open Proposal process. However, our overall goal was to improve proposals and provide rapid feedback to allow proposals to be retracted to reduce unnecessary investment of time. Every implementation resulted in revised proposals and some resulted in retracted proposals (Table 1). Through seven surveys sent out to proposers and commenters, we also learned that 13 (22%) out of 60 proposal authors who responded reported an impact on their team, such as adding new members and advisers. And, 71 (73%) of 97 total respondents viewed the process as “satisfactory” or “very satisfactory.”

But more than the numbers, the examples are illustrative. Through several forums we heard about a need for an online randomization tool to make it easier to do certain types of studies. Several comments supported the need for the randomization tool, but one commenter, while also supporting the need, wrote of two existing resources that he suggested might fit the bill. Within a day, the authors of the original proposal responded with further details on these existing tools, and concluded that the existing tools indeed met the need. They retracted their submission. This, we believe, was an example of a significant success.

### Opportunities, challenges and future growth

UCSF's CTSI is establishing a sustainable model to share the approach and tool. As we disseminate the approach, we share key lessons. Clearly, the culture of the group targeted makes a difference in the success of the approach. The first time that the central IT group at UCSF implemented this approach they achieved early and rapid engagement with multiple comments and iterated proposals. This group, we believe, was primed to use tools such as these for online engagement. Engaging the Department of Medicine in its call for proposals to fund registries was more difficult. Here culture certainly played a part—faculty had to get used to this novel approach—although some reticence will likely resolve with time and repeated exposure. In addition, because the Department of Medicine was funding its own group's ideas, the pool of those reviewing the proposals was constrained to the Department, thus constraining commenting. We wonder if in this type of scenario it might be more effective to open up the call to review the proposals to the broadest university community. In general, we believe that the largest potential group of commenters should be included. And in this, we have developed a growing appreciation for identifying incentives not only for proposers but also for commenters and reviewers.

In general, we believe we have engaged a much larger community in finding solutions to stymied research processes. The Open Proposals approach allows us to gain the best features of bottom‐up approaches to innovation while still allowing iterative improvement, fluid team formation, and full integration with existing programs and personnel. The software continues to improve and the process itself appears to be gaining traction with institutional leaders and with participants alike. Disciplined crowdsourcing may be a useful tool in advancing complex systems such as research support. In addition, we are now exploring how to extend the approach to improve the development of scientific ideas and research teams.

To view UCSF Open Proposals, see: http://open‐proposals.ucsf.edu

## Financial Support

This project and publication was supported by the National Center for Advancing Translational Sciences, National Institutes of Health, through **UCSF‐CTSI Grant Number UL1 TR000004.** Its contents are solely the responsibility of the authors and do not necessarily represent the official views of the NIH.

